# Pauper's Work

**Published:** 1889-06-29

**Authors:** 


### Paupers' Work.

One of the hardest things in the world is to feel that one's working days are done. However much we long for idleness in our prime, we do not like to think that we are fit for nothing in the way of labour. To cease from work of our own freewill is one thing, to be put on the shelf is another. Among those whom we somewhat narrowly define as the working classes this feeling is very strong. How often one hears the grandmother by the fireside fretting: "I'm no use here now; it's time I was out of the world." And this when their children are happy and proud to keep them in as much comfort as they can give, and their grandchildren cannot imagine what life would be without granny to run to in every childish joy or grief. Among the best class of those whom extreme poverty and lack of kin lead to the workhouse, the same complaint is to be heard. They long to work; to feel themselves not wholly dependent on charity. It is, therefore, a kindly thing of the guardians of the Paddington Workhouse to permit the inmates to do what work they can. A sale, which was held in the dining-room of the workhouse on Monday last, and was opened by Lady Randolph Churchill, showed what paupers, the flotsam and jetsam of the poor, can do. Much of the needlework was pretty and artistic, and the sewing of the useful articles was admirable. Some knitted hearthrugs had a very good effect, and represented a very large amount of labour. The show in general gave proof not only of willing industry on the part of the inmates, but of much kindness on the part of the visitors who had taught them the various kinds of work. Lady Randolph Churchill seemed both interested and touched by the display, and made extensive purchases. The proceeds of the sale are to be expended chiefly in the purchase of materials to continue the work, as well as to give small payments to the workers.

